# The impact of COVID-19 movement restrictions on physical activity in a low-income semi-rural population in Malaysia: A longitudinal study

**DOI:** 10.7189/jogh.11.05029

**Published:** 2021-12-25

**Authors:** Ruth Salway, Tin Tin Su, Roshidi Ismail, Miranda Elaine Glynis Armstrong, Charlie Foster, Laura Johnson

**Affiliations:** 1 Centre for Exercise, Nutrition, and Health Sciences, School for Policy Studies, University of Bristol, UK; 2 South East Asia Community Observatory (SEACO), and Global Public Health, Jeffrey Cheah School of Medicine and Health Sciences, Monash University, Malaysia

## Abstract

**Background:**

The COVID-19 pandemic prompted movement restrictions in countries worldwide, impacting on physical activity (PA), a major non-communicable disease risk factor, and thus may have unintentional long-term health implications. In semi-rural areas of low-middle-income-countries (LMICs), where occupational activity is the main source of PA, changes in PA associated with COVID-19 restrictions are unknown. We investigated the impact of Movement Control Order (MCO) restrictions in a semi-rural region of Malaysia.

**Methods:**

The South East Asia Community Observatory (SEACO) is a dynamic prospective community cohort. We contacted a random sample of 1007 adults (18+) who had previously provided PA data in 2018. We asked about PA during the MCO (March-May 2020) and at the time of interview (June 2020).

**Results:**

During the MCO, PA reduced by a mean of 6.7 hours/week (95% confidence interval (CI) = 5.3, 8.0) compared to 2018, with the largest reductions among those in employment. By June, PA was 3.4 hours/week (95% CI = 2.0, 4.8) less than 2018, leaving 34% of adults currently inactive (20% in 2018). Reductions in occupational PA were not replaced with active travel or activity at home. Despite these observed reductions, most participants did not think the MCO had affected their PA.

**Conclusions:**

Movement restrictions are associated with lower PA lasting beyond the period of strict restrictions; such longer-term reductions in PA may have a detrimental impact on health. Future MCOs should encourage people to be active, but may additionally need targeted messaging for those who don’t necessarily realise they are at risk. In particular, policies developed in more affluent countries may not easily translate to LMICs.

In March 2020 the World Health Organisation declared the COVID-19 outbreak as a global pandemic [1], with over 4.7 million deaths globally by the end of September 2021. Governments have reacted by instigating various measures to prevent virus transmission, most commonly by restricting population movement to different degrees.[2] Restrictions include limits on travel, indoor and outdoor gatherings, and non-essential work, and have impacted on the ability of all ages to move and participate in activities of everyday living, recreation, education and employment, thus indirectly affecting physical activity (PA),[3] with potentially important consequences for health.

Physical inactivity is one of the strongest determinants of non-communicable diseases (NCD), including cardiovascular disease, stroke, type 2 diabetes, and cancer, is estimated to cause over 5 million deaths worldwide annually, and is a target for change in the Global NCD action plan[4]. There may also be more immediate benefits of PA in preventing both COVID-19 hospitalisations and deaths [5]. Global studies have suggested a consistent decrease in population levels of PA [6] during the pandemic and understanding national changes in PA helps policy makers consider the unintentional consequences of COVID-19 mitigations and prioritise resources and programmes to manage the impact of restrictions, both in the return to normal and in event of future lockdown restrictions.

Stockwell et al [7] conducted a global systematic review of studies reporting changes in PA before and during COVID-19 lockdowns. The majority show a substantial decrease in PA during the pandemic across all reviewed populations. However, over 90% of the 64 studies included used cross-sectional designs with retrospective measures of pre-lockdown PA, often using study-specific PA measures which are not comparable across studies. Furthermore, difficulties in data collection during a pandemic meant that most were convenience samples, or lacked information on important confounders such as gender or socio-economic status. The authors also reported considerable uncertainty about the scale and impact of lockdown polices at national and local levels. They recommended that to assess the real impact of COVID-19 lockdown measures, future studies should use stronger causal designs, assess impacts before and after lockdown periods, and use validated self-report measures with detailed information about domain-specific PA types, intensities, and patterns.

Most of the evidence to date is from high-income countries in Europe, USA, Canada, and Australia (75% of studies in the systematic review). By contrast, the Association of South East Asian Nations comprises 10 South East Asian countries (9% of the world’s population) and yet there have been hardly any studies of the effects of the COVID-19 pandemic on PA in the general population in these countries. We found only one study that reported separately by country [8], reporting leisure PA only. These countries are distinct with different demographics, types, and patterns of PA, with high levels of occupational PA. Stockwell et al propose three possible reasons for the change in PA during lockdown: closure of sports and leisure facilities, government restrictions on time spent outdoors and allowed activities, and reductions in active travel due to workplace changes. However, this ignores those populations who gain most of their PA in occupational settings, for example manual labour, which contributes differentially by income [9]. Much of the current discussion around promoting PA during and after lockdowns has focused on leisure-time PA [7], and in particular the use of digital-based solutions (eg, smartphone apps and online fitness classes) to increase PA [10], but such solutions may be less accessible in these countries.

Malaysia is a low-middle income country (LMIC) in South-East Asia. The 2019 National Health and Morbidity Survey [11] estimated that 25% of the population were insufficiently active, with a slightly lower estimate of 20% in rural areas. On 16th March 2020, the Malaysian government introduced a national Movement Control Order (MCO), closing all non-essential businesses, restricting travel, and going out for essential shopping or medical needs only. Going outdoors for the exclusive purpose of PA was not allowed. Restrictions were eased from 5th May 2020 (conditional MCO; cMCO), and more areas opened up from 10th June (recovery MCO; rMCO), although sports facilities remained closed. By July 2021, Malaysia had reported a total of 952 000 COVID cases and 7440 deaths since 1st January 2020.

The South East Asia Community Observatory (SEACO) health and demographic surveillance system is a dynamic prospective community cohort in Segamat, a semi-rural region of Malaysia [12], whose primary industry is agriculture, followed by wholesale/trading and accommodation/food service industries [13]. In 2018, the SEACO health survey collected data on PA using the Global Physical Activity Questionnaire (GPAQ) [14]. For this study, we contacted 1000 randomly selected adults who had previously provided PA data and asked them about their PA during the MCO and the rMCO. Thus, this longitudinal study includes validated contemporaneous PA measures pre and post lockdown, as well as data on relevant confounders. This is an exploratory analysis to describe the effect of COVID-19 lockdown in a semi-rural population in a LMIC country, to see first if PA declined and second to explore how any changes in PA differed by specific domains.

## METHODS

### Design and participants

**Figure 1:**
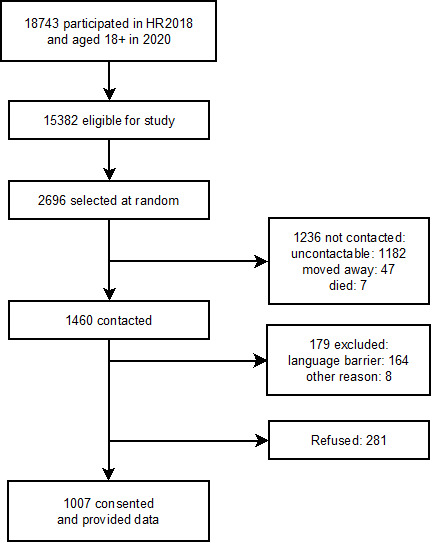
Flow diagram of participants.

SEACO is a dynamic prospective community cohort of 13 335 households surveyed yearly since 2012 in Segamat, a semi-rural region of Malaysia, which includes questionnaire survey, blood tests, and physical measurements [12]. The SEACO Health Round Survey 2018 (HR2018) took place between July 2018 and August 2019 and included data on PA, measured using the GPAQ, which has been validated in Malaysian adults [14,15]. A project using existing SEACO data (Risk factor surveillance update; Project ID: 13142) received ethical approval on 13th June 2018 and further ethical approval was obtained from the Monash University Human Research Ethics Committee (Project ID: 13142 approved 5th June 2020) to amend this to accommodate a special COVID-19 telephone survey to collect longitudinal data on PA. All adults aged 18 or over who participated in HR2018 and reported GPAQ data (over 99%), and for whom a contact telephone number was held were eligible for the COVID-19 survey (n = 15 382). Participants were recruited until the target sample size of 1000 was met, by selecting at random from the list of HR2018 participants, using simple random sampling in Stata v15. In total, we attempted to contact 2696 participants with 1288 contacted between 30th June 2020 and 29th July 2020 (1408 uncontactable, moved, deceased, or excluded due to language barrier). Of these, 1007 (78%) provided consent via telephone (**Figure 1**). Trained data collectors from SEACO conducted the interview (median duration 21 minutes; inter-quartile range: 13.4 minutes) following SEACO’s telephone interview protocol, with responses entered into an electronic handheld device during the interview. Participant’s identity was confirmed using name and National Registration Identity Card Number.

### Measurements

#### Physical activity

PA was recorded at three time points: in 2018/19 (pre-pandemic) as part of the HR2018; retrospectively during the strict lockdown MCO (January-May 2020) and at time of interview (during the rMCO June-July 2020). PA was assessed using GPAQ, and data processed according to WHO protocol l [16] to derive estimates of the average number of minutes per week engaged in PA in three domains (occupational, transport and leisure) and total PA at each time point. We classified participants as insufficiently active if they engaged in less than 600 MET-minutes of PA per week [16]. The main analyses use continuous longitudinal measures of change in minutes of PA from baseline (pre-COVID), to reflect the focus of the paper on change in PA. In addition, the use of a continuous measure is more sensitive to change than a binary indicator, with the latter having less power to detect differences [17].

#### Other data

Demographic data on age, sex, and ethnicity (Malay, Chinese, Other (Indian, Orang Asli and other ethnicities, grouped due to small numbers)) were self-reported in HR2018, along with highest education level obtained (up to primary, secondary, tertiary) and employment status, recoded into ‘Working’ (including full-time work, part-time work and casual work) and ‘Not Working’ (looking after the home, students, pensioners, other). Participant’s height and weight were measured in HR2018, and BMI was derived and classified as up to healthy weight (≤25 kg/m^2^), overweight (>25 kg/m^2^ and <30 kg/m^2^), and obese (≥30 kg/m^2^) [18]. Those aged over 35 were asked if a doctor had diagnosed hypertension or diabetes; these were combined to form a variable indicating the presence of a doctor-diagnosed NCD.

During the telephone interview, we asked participants to confirm their sex and age, and asked them their current employment status. As well as the GPAQ measures of PA, we asked participants if they felt they were less physically active during the MCO (responses to a great extent, somewhat, very little, or not at all). We also asked about confirmed and suspected COVID-19 infection, and asked about any changes in their job (working at home, reduced hours, stopped working, lost job, didn’t go to work).

### Statistical analysis

A sample size of 1000 was calculated to have 80% power to detect a minimum reduction of 0.7 hours/week in total PA (8% of HR2018 levels) at a 5% significance level, or an increase in inactivity of 3%-5%. We compared demographics between the study sample and the full HR2018 sample of 18743, and reported levels of missing data. We summarised PA in hours/week for each domain (occupational, transport, and leisure) and overall by each time point. As PA estimates are heavily right skewed, we reported medians and interquartile ranges (IQR). The number of participants engaging in PA due to transport or leisure is small in this population, so we additionally reported the percentage of participants who engaged in any minutes of activity in these domains.

We calculated the difference in total PA (hours/week) between baseline and MCO, and baseline and rMCO for each participant, and reported estimates of the mean and confidence intervals overall and by sex, ethnicity, employment status, education, BMI category, and doctor-diagnosed NCD (over 35-year-olds only). While the PA estimates at each time point were heavily skewed, the differences were approximately normally distributed, so we fitted a linear regression model for the PA difference, and included both demographic variables (age, sex, ethnicity) and socioeconomic variables (baseline employment status and education). We repeated the model to additionally include the health risk indicators, BMI category and doctor-diagnosed NCD; this was available for participants aged over 35 in 2018 only. As changes in PA can depend on initial PA levels, we repeated the NCD analysis adjusting for baseline PA in 2018 as a sensitivity analysis. Finally, we explored participants’ perceptions of their PA during the MCO by fitting a logistic model for those who reported that they were less physically active (either to a great extent or somewhat), including the demographic and SES variables above and any reported changes in their job during the MCO.

All analysis was done in Stata v15 [19], and residuals were inspected to check deviations from model assumptions. A very small number of participants belonged to the same household and so all confidence intervals and models used robust standard errors to adjust for within-household clustering. As this is an exploratory analysis, out interpretation is based on the magnitude and 95% confidence intervals of estimates, rather than on statistical significance.

## RESULTS

The 1007 consenting participants were demographically similar to the SEACO cohort as a whole (Table S1 in the **Online Supplementary Document**), with low levels of missing data (0%-1% for individual questions; 2% missing GPAQ data at any time point). COVID-19 infection in the sample was low, with only 3 participants (0.3%) reporting either a positive test or suspected infection.

**Figure 2:**
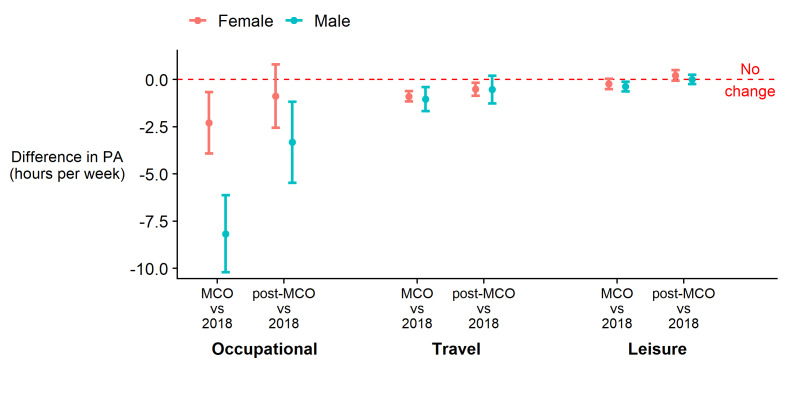
Percentage of participants classified as insufficiently active at each time point by sex.

**Figure 3:**
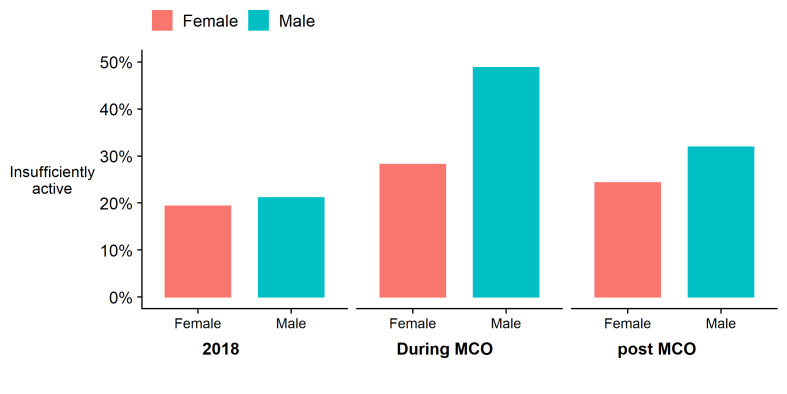
Average change in PA (hours/week) between 2018 and MCO, and between 2018 and rMCO, by domain and sex, with 95% confidence intervals.

In 2018, 20% of the sample were insufficiently active, with a median PA of 14.0 hours/week (IQR = 26.5 hours/week). During the MCO this halved to a median 7.0 hours/week (IQR = 18.0 hours/week) with 37% classed as insufficiently active (**Figure 2**; Table S2 in the Online Supplementary Document), and during the rMCO, a median of 9.0 hours/week (IQR = 20.8 hours/week) with 28% insufficiently active. At all three time points, occupational PA was the largest contributor to total PA. Median weekly PA for transport and leisure domains were zero (Table S2 in the **Online Supplementary Document**) for all groups and all time periods, meaning that over 50% engaged in no PA in the respective domain. Reductions in volume of PA were seen in all three domains (Figure 3). Participation in transport PA reduced from 25% in 2018 to 8% during the MCO (rMCO = 14%). Participation in leisure PA increased during the rMCO, although the average time spent was lower, and there were no differences in participation between those who did and did not experience a reduction in occupational PA (Table S3 in the **Online Supplementary Document**).

Overall, the estimated mean change in PA between 2018 and the MCO was a reduction of 6.2 hours/week (95% CI = 4.8, 7.5) with a 2.4 hours/week (95% CI = 0.9, 3.8) reduction during the rMCO (**Table 1**). We saw similar patterns - large reductions during the MCO and smaller reductions in the rMCO – for all sexes, ethnicities, and employment status. The reduction in PA was nearly three times greater for men than for women, and eight times greater among those in employment than those not working. PA among those in employment reduced by half during the MCO for an average difference of 11.3 hours/week (95% CI = 9.1, 13.6), and while it increased during the rMCO, it was still lower than 2018 by an average of 5.6 hours/week (95% CI = 3.2, 8.0). The regression model (Table 2) found that on average, the PA of a 50-year old primary-educated working Malay man decreased by 13.8 hours/week (95% CI = 10.1, 17.5) during the MCO and by 6.7 hours/week (95% CI = 2.8, 10.5) during the rMCO. For a similar woman, PA reduced by 9.1 hours/week (95% CI = 5.4, 12.7) and 4.8 hours/week (95% CI = 0.9, 8.8) respectively. Univariate associations of employment status and ethnicity with change in PA remained after adjusting for each other, with larger decreases among men, those in employment, and those of Chinese ethnicity. Among over 35-year-olds, PA decreased to a lesser degree for those with a doctor-diagnosed NCD compared to those without (Table S4 in the **Online Supplementary Document**), but this attenuated completely when adjusting for baseline PA (Table S5 in the **Online Supplementary Document**). Model assumptions were checked by visual inspection of the residuals of the models, and revealed no issues with model suitability.

**Table 1:** Estimates and 95% confidence intervals for change in PA (hours/week) by demographics

	Difference between 2018/19 and MCO (hours/week)			Difference between 2018/19 and rMCO (hours/week)		
	**Mean**	**95% Confidence interval**	*P*-value*	**Mean**	**95% Confidence interval**	*P*-value*
**All**	-6.2	(-7.5, -4.8)	<0.0005	-2.4	(-3.8, -0.9)	0.001
**Sex:**						
Male	-9.6	(-11.7, -7.4)	<0.0005	-3.9	(-6.2, -1.5)	0.001
Female	-3.4	(-5.1, -1.8)	<0.0005	-1.2	(-2.9, 0.6)	0.193
**Ethnicity:**						
Malay	-5.9	(-7.6, -4.2)	<0.0005	-1.6	(-3.5, 0.2)	0.077
Chinese	-8.9	(-11.9, -5.9)	<0.0005	-5.8	(-8.7, -2.9)	<0.0005
Other	-3.4	(-7.0, 0.2)	0.068	-0.5	(-4.1, 3.1)	0.795
**Employment status:**						
Working	-11.3	(-13.6, -9.1)	<0.0005	-5.6	(-8.0, -3.2)	<0.0005
Not working	-1.4	(-2.8, 0.0)	0.056	0.6	(-0.9, 2.2)	0.439
**Highest education:**						
Up to primary	-5.6	(-8.0, -3.3)	<0.0005	-2.2	(-4.6, 0.2)	0.076
Secondary	-6.8	(-8.8, -4.9)	<0.0005	-2.9	(-5.0, -0.8)	0.007
Tertiary	-6.0	(-9.7, -2.3)	0.002	-1.2	(-5.2, 2.8)	0.559
**BMI category (2018):**						
Underweight/Healthy	-7.0	(-9.2, -4.9)	<0.0005	-3.5	(-5.8, -1.1)	0.004
Overweight	-7.5	(-10,1, -4.9)	<0.0005	-3.2	(-5.9, -0.6)	0.018
Obese	-4.1	(-6.5, -1.7)	0.001	-0.3	(-2.8, 2.2)	0.810
**Doctor-diagnosed diabetes/hypertension:^†^**						
No	-7.6	(-9.6, -5.6)	<0.0005	-2.9	(-4.9, -0.8)	0.006
Yes	-2.5	(-4.5, -0.5)	0.015	0.0	(-2.1, 2.2)	0.985

MCO – Movement Control Order (March – May 2020). rMCO – recovery MCO (June 2020). BMI – body mass index

*For a hypothesis test of no difference in PA.

†Those aged 35 and over only.

**Table 2:** Modelled difference in change in PA (hours/week)

	Difference in PA (hours/week) between 2018/19 and MCO, N = 933			Difference in PA (hours/week) between 2018/19 and rMCO, N = 932		
	Estimate	95% CI	*P* value*	Estimate	95% CI	*P* value*
Intercept^†^	-13.8	(-17.5, -10.1)	<0.0005	-6.7	(-10.5, -2.8)	<0.0005
**Age** (per 10 years)	1.5	(0.5, 2.5)	0.004	1.3	(0.1, 2.4)	0.028
**Sex:**						
Male	Reference			Reference		
Female	4.8	(1.8, 7.8)	0.002	1.8	(-1.6, 5.2)	0.288
**Ethnicity:**						
Malay	Reference			Reference		
Chinese	-4.2	(-7.6, -0.8)		-5.0	(-8.6, -1.5)	
Other	4.4	(0.2, 8.6)	0.001	3.3	(-1.0, 7.6)	0.002
**Employment status:**						
Working	Reference			Reference		
Not working	7.5	(4.7, 10.3)	<0.0005	4.8	(1.5, 8.1)	0.005
**Highest education:**						
Up to primary	Reference			Reference		
Secondary	1.5	(-1.7, 4.7)		1.5	(-1.8, 4.9)	
Tertiary	2.9	(-1.5, 7.4)	0.404	3.6	(-1.3, 8.5)	0.329

PA – physical activity. MCO – Movement Control Order (March – May 2020). rMCO – recovery MCO (June 2020)

*P-value refers to test for the regression coefficient equal to zero for age and for a difference between categories for the categorical variables (sex, ethnicity, employment status and highest education)

†Intercept represents average hours/week change in PA for a 50-y old primary-educated working Malay man, of healthy weight.

**Figure 4:**
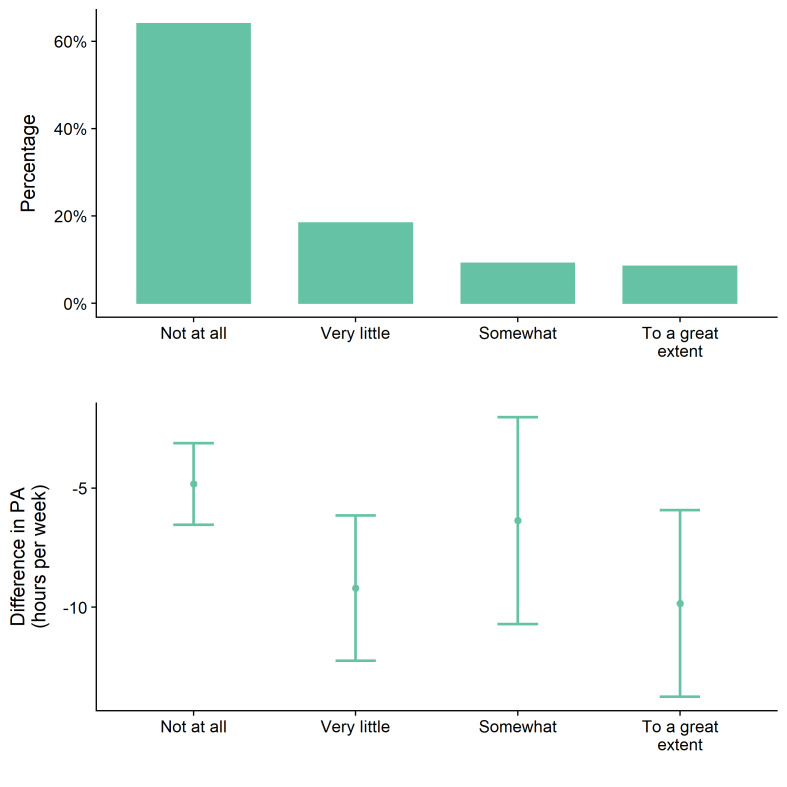
Participant’s perception of change in PA (“*Because of the MCO, I have been less physically active…*”) and actual average change in PA (with 95% confidence intervals).

Only 18% of participants reported that they felt they had been less active during in the MCO (**Figure 4**). A logistic regression model for perceived reductions in PA found that perceptions did not differ by actual PA, employment status, ethnicity, or education (Table S6 in the **Online Supplementary Document**). Just under a third (30%) reported changes in working habits during the MCO, with the most common being not going to work (33%) but this was not associated with perceptions of activity reduction.

## DISCUSSION

We have shown for the first time that COVID-19 movement restrictions more than halved the time spent physically active in a semi-rural LMIC, specifically by limiting occupational PA. Current policies mitigating the unintentional consequences of lockdowns on PA focus on transport and leisure-time PA whereas our findings highlight the need for strategies to compensate for lost occupational activity in populations where that is how they are primarily active. A fifth of adults were classified as insufficiently active before the COVID-19 pandemic, consistent with the NHMS 2019 [11] estimate for rural areas. This rose to over a third during the MCO, with 2 in 7 adults still insufficiently active during the rMCO. This reduction in PA is in line with decreases seen in other countries with restrictions during the COVID-19 pandemic [7,8,20], eg, reductions in daily step counts of 40%-50% [3]. However, most previous evidence is from high-income countries, and this is the first study to report a reduction in domain-specific PA in a Southeast Asian adult population.

PA decreased by a mean of 6.2 hours/week during the MCO, compared to 2018. During the less restrictive rMCO (which included the opening of many businesses, health and well-being, childcare, swimming, and leisure facilities, and allowed interstate travel, religious worship, meetings & exhibitions), PA increased, but did not return to 2018 levels and still showed an overall reduction of 2.4 hours/week. This is especially of note since Malaysia has been subject to some form of MCO continuously since March 2020, with the rMCO period covered here being the least restrictive. Since then, there have been two further strict MCOs, with the most recent extended indefinitely as of 15th August 2021. Our results suggest that the impact of MCOs on PA is not limited to periods of strict restrictions, and that continued restrictions may have longer term impact.

Most notably, in this study the largest contributor to total PA is from the occupational domain, with only 18% of respondents engaging in leisure PA and 25% in PA for transport before the pandemic. We saw reductions in total PA across all three domains, although participation in leisure PA increased during the rMCO, with the largest reductions in occupational PA. The biggest employment sectors in Segamat are agriculture, wholesale, and food service industries, with many considered essential services during the pandemic. Unlike high income countries, where people often worked from home during restrictions (eg, 47% in the UK [21]) only 15% of employed adults in our study reported working from home, and the majority (70%) experienced no changes to their job. However, we saw reductions in occupational PA even for those who reported no change in job circumstances, suggesting other aspects of the work environment, for example as a result of COVID-secure measures, may have impacted on PA. We saw no evidence that a reduction in occupational PA was replaced by increased PA in another domain. Few studies of PA during the pandemic have distinguished between the different domains (occupational, transport, and sport/leisure) and evidence is inconsistent. A study in Greece [22] found reductions in occupational and transport PA, but increases in leisure PA, whereas a Japanese study of predominantly sedentary office workers [23] found the opposite. It is thus important to consider the impact of movement restrictions on the different PA domains separately, as this impact may differ depending on the population.

Despite the large observed decreases in PA, participants’ perception did not reflect this, with the majority stating that the MCO had not affected their PA, and perceptions were not associated with actual changes in PA. This is important, as much of our knowledge of the impact of the pandemic comes from surveys initiated after the start of the pandemic that ask about perceived changes in various behaviours [24]. A strength of our study is the use of an ongoing surveillance study to measure actual change, and we found large reductions in PA which would not have been evident if we had relied on perceptions or retrospective measures of pre-COVID activity. It is therefore important for governments to invest in surveillance surveys to be able to accurately understand the impact of atypical events on health in future.

Our study has a number of strengths, especially in light of the difficulties of conducting research in a pandemic. Research to date on the impacts of the pandemic has relied on online surveys and recruitment via social media, which tends to over-recruit participants from high-income urban regions. By contrast, our study population is a semi-rural low-income area of Malaysia, and our use of telephone interviews allowed us to reach a population who are typically difficult to capture and are under-represented in research on the impact of the pandemic. Our longitudinal data has pre-COVID estimates of PA measured at the time rather than retrospectively, and the use of a validated questionnaire in GPAQ allows comparability with other studies; although self-report measures are not sufficiently accurate for individual assessment of PA, GPAQ has been shown to be appropriate for population estimate and monitoring change in PA in a population sample of this type [25]. While there may be some potential bias in the estimates of current PA due to the pandemic itself, we note that first, this is likely to be small as the majority of participants reported that they did not feel they had been less active since the pandemic, and second that, given the extremely large reported differences in PA, such bias is unlikely to be substantial enough to affect our conclusions. Restricting to those with telephones may introduce selection bias, although our sample was found to be broadly demographically similar to the SEACO population as a whole, with slight under-representation of the very lowest education category (note that education is adjusted for in the statistical models). The SEACO population generally has a smaller proportion of young and middle-aged adults, and fewer of Chinese ethnicity, compared to the district as a whole [12], although the income and socio-economic distributions are very similar. In addition, we relied on retrospective self-report of PA during the MCO itself, although the differences between the rMCO and 2018 are based on contemporaneous measures. We were also limited in data about the nature of the respondent’s employment, and so were unable to explore industry differences in reductions in PA.

It is important to understand different PA patterns, as well as overall totals of PA, especially in populations less well-represented in the current literature as these can reflect quite different impacts on health and thus policy needs. It is not clear whether the mismatch between perception of PA and reality is specific to those for whom PA is a product of daily work activities, rather than a voluntary/leisure behaviour in its own right. However, it does suggest that, for some groups at least, the true impact may be underestimated by surveys conducted after the onset of the pandemic. Future studies should therefore avoid relying on perceptions of change or retrospective measures of activity, either in assessing the impact of COVID-19 or in planning for future MCOs, and governments need to invest in surveillance data to be able to reliably monitor changes in public health over time, especially in response to major events such as the pandemic. Future MCOs should consider how restrictions affect PA and include options to encourage people to remain active, but may additionally need targeted messaging for those who don’t necessarily realise they are at risk. In particular, policies to promote PA during restrictions in more affluent countries, such as maintenance of leisure PA or the use of digital solutions, may not be easily translatable to LMICs, leaving these countries at higher risk of health consequences in future. The policy priority should remain that in an acute phase of restrictions, transport and recreational PA should be presented as a means to mitigate some of the reduction in occupational activity, with some access to resources outside the home still allowed.

## CONCLUSION

Movement restrictions due to the COVID-19 pandemic are associated with large reductions in PA, especially in occupational PA, lasting beyond the period of strict restrictions. Future MCOs should include options to encourage people to be active, but may additionally need targeted messaging for those who don’t necessarily realise they are at risk. In particular, policies in more affluent countries, such as maintenance of leisure PA or the use of digital solutions, may not easily translate to LMICs.

## Additional material


Online Supplementary Document
