# Supplementary material for: The impact of COVID-19 movement restrictions on physical activity in a low-income semi-rural population in Malaysia: A longitudinal study
Source: J Glob Health. 2021 Dec 25;11:05029. doi: 10.7189/jogh.11.05029 (PMC8710227; doi:10.7189/jogh.11.05029)
Supplement: Online Supplementary Document [file jogh-11-05029-s001.pdf]

Supplementary Material for 'The impact of COVID-19 movement restrictions on physical activity in a low-income semi-rural population in Malaysia: a longitudinal study'

Ruth Salway, PhD[1], Tin Tin Su[2], Roshidi Ismail[2], Miranda Elaine Glynis Armstrong, PhD[1], Charlie Foster, PhD[1], Laura Johnson, PhD[1]

Table of Contents

Table S1: Comparison of 2018 Demographics and missing data 2

Table S2: Hours of PA by domain and % insufficiently active at all three time points 3

Table S3: Percentage engaging in transport or leisure PA for those with and without a reduction in occupational PA 4

Table S4: Modelled difference in change in PA (hours/week) including NCD and BMI category 5

Table S5: Modelled difference in change in PA (hours/week) including NCD and BMI category, adjusting for baseline PA in 2018 6

Table S6: Modelled perception of change in PA - logistic model for the probability that the participant felt they were less active during the MCO 7

Table S1: Comparison of 2018 Demographics and missing data

	HR2018		COVID-19 Survey		
	N (%) Mean (sd)		N (%) Mean(sd)		Missing data N (%)
**All**	18743		1007		
**Sex**					0 (0%)
Male	8251	44%	456	45%	
Female	10492	56%	551	55%	
Age in 2018	49.0	(18.5)	49.5	(16.5)	13 (1%)
**Employment status (2018)**					0 (0%)
Working	8245	44%	489	49%	
Full-time	5144	27%	290	29%	
Part-time	796	4%	44	4%	
Self-employed	2261	12%	153	15%	
casual worker	44	<0.5%	2	<0.5%	
Not working	10497	56%	481	51%	
Homemaker	5341	29%	271	27%	
Not working	3136	17%	132	13%	
Pensioner	916	5%	74	7%	
Student	1104	6%	4	4%	
**Ethnicity**					0 (0%)
Malay	12049	64%	653	65%	
Chinese	4417	24%	214	21%	
Indian	1842	10%	105	10%	
Orang Asli	358	2%	18	2%	
Other	76	<0.5%	17	2%	
**Physical activity**					
PA hours/week (2018)	18.5	(19.4)	18.5	(18.2)	3 (<1%)
% Insufficiently active (2018)	3976	22%	202	20%	3 (<1%)
Missing PA at any timepoint					20 (2%)

Table S2: Hours of PA by domain and % insufficiently active at all three time points

	2018/19 N=1002		During MCO N=991		During rMCO N=987	
	**median**	**IQR**	**median**	**IQR**	**median**	**IQR**
Occupational (hours)	12.0	26.5	6.0	14.4	7.0	20.5
Transport (hours)	0	0	0	0	0	0
% any transport[1]		25%		8%		14%
Nonzero transport (hours)	3.0	4.8	1.9	6.3	2.0	3.9
Sport/leisure (hours)	0	0	0	0	0	0.5
% any leisure[1]		18%		16%		28%
Nonzero leisure (hours)	3.0	3.5	2.0	2.5	2.0	3.0
Total (hours)	14.0	24.5	7.0	18.0	9.0	20.8
% Insufficiently active		20%		37%		28%

[1] more than 10 minutes of PA per day reported

Table S3: Percentage engaging in transport or leisure PA for those who did and did not experience a reduction in occupational PA

	Any transport PA[1]		Any leisure PA[1]	
	%	95% CI	%	95% CI
During MCO				
Reduction in occupational PA	8%	(6%, 10%)	18%	(14%, 21%)
No reduction in occupational PA	8%	(6%, 11%)	15%	(12%, 19%)
During rMCO				
Reduction in occupational PA	13%	(10%, 16%)	28%	(24%, 32%)
No reduction in occupational PA	16%	(13%, 19%)	28%	(24%, 32%)

[1] more than 10 minutes of PA per day reported

Table S4: Modelled difference in change in PA (hours/week) including NCD and BMI category

Only those aged 35 and over were asked about NCD so this table is based on a smaller sample size than Table 4

	Difference in PA (hours/week) between 2018/19 and MCO N=758			Difference in PA (hours/week) between 2018/19 and rMCO N=757		
	**Estimate**	**95% CI**	**p-value**	**Estimate**	**95% CI**	**p-value**
Intercept[1]	-16.4	(-21.5, -11.2)	<0.0005	-7.7	(-13.4, -2.0)	0.008
Age (per 10 years)	1.0	(-0.5, 2.8)	0.198	0.7	(-1.1, 2.4)	0.451
Sex						
Male	Reference			Reference		
Female	3.9	(0.5, 7.3)	0.025	1.4	(-2.3, 5.1)	0.458
Ethnicity						
Malay	Reference			Reference		
Chinese	-2.7	(-6.1, 0.8)		-3.4	(-7.0, 0.3)	
Other	5.7	(1.1, 10.3)	0.004[2]	4.4	(-0.4, 9.2)	0.011[2]
Employment Status						
Working	Reference			Reference		
Not working	6.3	(3.1, 9.5)	<0.0005	2.5	(-1.1, 6.0)	0.178
Highest education						
Up to primary	Reference			Reference		
Secondary	2.1	(-1.5, 5.8)		1.7	(-2.2, 5.6)	
Tertiary	4.4	(0.8, 9.5)	0.222[2]	3.5	(-2.1, 9.1)	0.332[2]
BMI category						
Underweight/Healthy	Reference			Reference		
Overweight	1.2	(-2.4, 4.7)		1.3	(-2.6, 5.1)	
Obese	2.8	(-0.9, 6.6)	0.330[2]	3.4	(-0.8, 7.6)	0.266[2]
Doctor diagnosed NCD						
No	Reference			Reference		
Yes	3.7	(0.6, 6.7)	0.017	2.2	(-1.2, 5.6)	0.198

[1] intercept represents average hours/week change in PA for a 50-year old primary-educated working Malay man, of healthy weight and with no doctor-diagnosed NCDs.

[2] p-value refers to test for a difference between categories

Table S5: Modelled difference in change in PA (hours/week) including NCD and BMI category, adjusting for baseline PA in 2018

Only those aged 35 and over were asked about NCD so this table is based on a smaller sample size than Table 4

	Difference in PA (hours/week) between 2018/19 and MCO (N=758)			Difference in PA (Hours/week) between 2018/19 and rMCO (N=757)		
	Estimate	**95% CI**	**p-value**	**Estimate**	**95% CI**	**p-value**
Intercept[1]	-9.4	(-13.2, -5.7)	<0.0005	-1.2	(-5.6, 3.3)	0.609
Baseline PA (per 1h)	-0.9	(-0.9, -0.8)	<0.0005	-0.8	(-0.9, -0.7)	<0.0005
Age (per 10 years)	-0.3	(-1.4, 0.9)	0.655	-0.7	(-2.1, 0.6)	0.289
Sex						
Male	Reference			Reference		
Female	5.1	(2.6, 7.6)	<0.0005	2.6	(-0.4, 5.6)	0.089
Ethnicity						
Malay	Reference			Reference		
Chinese	-2.3	(-4.7, 0.1)		-2.9	(-5.9, -0.3)	
Other	6.6	(2.8, 10.4)	<0.0005[2]	5.2	(1.1, 9.3)	0.002[2]
Employment Status						
Working	Reference			Reference		
Not working	-2.3	(-4.7, 0.1)	0.061	-5.3	(-8.3, -2.4)	<0.0005
Highest education						
Up to primary	Reference			Reference		
Secondary	3.2	(0.6, 5.7)		2.5	(-0.6, 5.5)	
Tertiary	1.2	(-2.5, 4.8)	0.048[2]	0.4	(-4.0, 4.8)	0.236[2]
BMI category						
Underweight/Healthy	Reference			Reference		
Overweight	-0.7	(-3.4, 1.9)		-0.5	(-3.7, 2.7)	
Obese	0.7	(-2.1, 3.6)	0.575[2]	1.3	(-2.2, 4.8)	0.526[2]
Doctor diagnosed NCD						
No	Reference			Reference		
Yes	0.2	(-2.1, 2.5)	0.862	-0.9	(-3.7, 1.8)	0.516

[1] intercept represents average hours/week change in PA for a 50-year old primary-educated working Malay man, of healthy weight and with no doctor-diagnosed NCDs, who engaged in a mean of 18.5 hours/week of PA in 2018.

[2] p-value refers to test for a difference between categories

Table S6: Modelled perception of change in PA - logistic model for the probability that the participant felt they were less active during the MCO (N=930)

	Less active in MCO		
	**Odds Ratio**	**95% CI**	**p-value**
Age (per 10 years)	0.83	(0.73, 0.95)	0.007
Sex			
Male	1		
Female	0.70	(0.47, 1.03)	0.070
Ethnicity			
Malay	1		
Chinese	1.00	(0.63, 1.59)	
Other	1.48	(0.89, 2.47)	0.310
Employment Status			
Working	1		
Not working	0.94	(0.62, 1.45)	0.794
Highest education			
Up to primary	1		
Secondary	1.12	(0.72, 1.75)	
Tertiary	1.15	(0.62, 2.14)	0.862
Job changes during MCO			
None	1		
One or more[1]	1.12	(0.75, 1.68)	
PA (increase of 1 hour/week)			
In 2018/19	1.00	(0.99, 1.01)	0.537
During MCO	0.99	(0.97, 1.00)	0.104
During rMCO	1.00	(0.99, 1.01)	0.736

[1] worked from home, reduced hours, stopped working, lost job, didn't go to work
